# Chicago Dental Society

**Published:** 1894-04

**Authors:** H. A. Costner

**Affiliations:** Cor. Sec.


CHICAGO DENTAL SOCIETY.—At the annual meeting of the Chicago Dental Society, held in Kindergarten Hall, April 3rd, 1894, the following officers were duly elected for the ensuing year. J. H. Wolley, president; C. E. Bentley, 1st vice-president; D. M. Gallie, 2nd vice-president; H. H. Peck, recording secretary; H. A. Costner, corresponding secretary; E. D. Swain, Treasurer; J. J. Whaley, Librarian; J. N. Crouse, director.

Yours truly,
H. A. COSTNER, Cor. Sec.

